# U.S. Healthcare Insurance Market Concentration from 2001 to 2016: Increased Growth in Direct Written Premiums and Overall Decreased Market Consolidation

**DOI:** 10.7759/cureus.7491

**Published:** 2020-03-31

**Authors:** Mitchell K Ng, Kenneth K Ng, Simon Song, Ahmed K Emara, Jason Ngo, Anooj Patel, Nihar Shah, Elias Mossialos, Sebastian Salas-Vega, Michael Mont, Nicolas Piuzzi

**Affiliations:** 1 Orthopaedic Surgery, Maimonides Medical Center, Brooklyn, USA; 2 Anesthesiology, State University of New York Downstate Medical Center, Brooklyn, USA; 3 Biomedical Engineering, Duke University, Durham, USA; 4 Orthopaedic Surgery, Cleveland Clinic Foundation, Cleveland, USA; 5 Anesthesiology, Saint Barnabas Medical Center, Livingston, USA; 6 Plastic Surgery, Case Western Reserve University School of Medicine, Cleveland, USA; 7 Orthopaedics, Case Western Reserve University School of Medicine, Cleveland, USA; 8 Health Policy and Economics, The London School of Economics and Political Science, London, GBR; 9 Orthopaedic Surgery, Lenox Hill Hospital, New York, USA; 10 Orthopaedic Surgery, Cleveland Clinic, Cleveland, USA

**Keywords:** healthcare insurance, market consolidation, direct written premiums, herfindahl-hirschman index

## Abstract

With the establishment of state-based health insurance marketplaces, how U.S. health insurers are responding to market pressures and influencing premiums have represented important questions. We made novel use of the Standard and Poor’s (S&P) Financial, a Wall Street financial dataset platform, to analyze trends in market capitalization and total direct written premiums (DWPs) from 2001 to 2016 of the top 5, 10, and 25 health insurance companies. Our results indicate that the market concentration of publicly traded companies has remained relatively stable over the past decade. The top 5, 10, and 25 health insurance companies were 43.5%, 57.5%, and 78.6% of the total market share in 2001 and 39.4%, 52.9%, and 72.8% in 2016, respectively. DWPs have grown nearly four-fold from $177 billion to $631 billion at a compounded annual rate of 8.8%, consistent with overall healthcare sector growth. Aggregating state-specific data, the overall U.S. health insurance market has become slightly less consolidated over recent years, as measured using the population-weighted Herfindahl-Hirschman index, a measure for market concentration, falling from 3,817 to 2,174 during this time period. As health insurance costs place a growing burden on American families, additional efforts are needed to study the impact on choice, quality, access, cost, and value to patients and providers from evolving health insurance markets.

## Introduction

Changes in the health insurance market can profoundly affect patients, payers, and providers [1-3]. From a patient’s and payer’s perspective, health insurer concentration may decrease health plan choice and increase premiums [4,5]. For providers, it may also reduce bargaining power in reimbursement and autonomy in practice [6]. Especially with the passage of the Affordable Care Act (ACA) and the establishment of state-based health insurance marketplaces (HIMs) [7], how health insurers are responding to market pressures and influencing premiums have therefore represented important questions in the U.S. healthcare sector.

These issues are, however, challenging to examine on a national scale, in part due to the difficulty in obtaining comprehensive data for the multitude of insurers that exist [5,6]. Citing calls by policymakers for additional evidence, a recent study found that 57% of U.S. metropolitan statistical areas were highly concentrated for insurers in 2016, with mean measures of market concentration declining by 1% between 2011 and 2016 [8].

However, it may be difficult to interpret the significance of these findings. Historical and insurance premium data are needed to thoroughly assess the impact of recent health insurance reforms. The ACA also gives states wide discretion on the implementation of premium rating requirements in their individual health insurance markets, resulting in state-specific geographic rating areas [9,10]. To therefore consider how market concentration has changed with recent policy reforms and affected premiums, the recent evidence should be compared with analyses that are based on whole state rating areas and incorporate longer-term market trends and health insurance premium data.

To help address these gaps, we made novel use of a financial dataset, Standard and Poor’s (S&P) Financial LLC, to analyze long-term trends in market concentration and total direct written premiums (DWPs) corresponding to the overall U.S. health insurance market from 2001 to 2016. We tracked market share of the top 5, 10, and 25 health insurance companies by revenue from 2001 to 2016, quantified growth of DWPs over this time period, and measured long-term changes in health insurance market concentration at a state level over this period of interest.

## Materials and methods

This study used data from S&P Financial (Standard & Poor’s Global Market Intelligence), a platform used widely by Wall Street for its market data, to obtain DWPs for U.S. health insurance companies. Covering 99.9% of the world’s market capital, S&P Financial covers more than one million transactions and provides sector-specific financial data for 88,000 publicly listed and 825,000 private companies worldwide.

Data extraction

Insurance statutory financials were screened using S&P Financial’s DataWizard tool for the following criteria: (i) filter types - health; (ii) operating status - operating, acquired/defunct; (iii) reporting level - S&P groups/unaffiliated companies; (iv) entity types - companies. Yearly data was obtained using the following parameters: (i) period (e.g., 2001Q1-2016Q4); (ii) line of business - annual revenue (total health); (iii) geography - state (North American Industry Classification System).

Total direct written premiums

Annual revenues based on DWPs (billion dollars) for each health insurance company (publicly listed and private) were calculated for each year between 2001 and 2016. The total market size was estimated based on the 716 unique insurance companies that were available in the database by summing the DWPs for each year. Annual market shares were then calculated for the 5, 10, and 25 largest companies by revenue and market capitalization over the period of interest relative to the total market size. For the publicly traded U.S. health insurers, total market capitalization (billion dollars) was collated and analyzed to determine the top 5, 10, and 25 companies annually throughout the period of interest. Of note, we chose to determine market share by total DWPs and not market capitalization, as private companies are not obligated to release all data, and the best way to value them is through projected revenues over a timespan.

Health insurance market concentration

Market concentration was measured at a state level using the Herfindahl-Hirschman Index (HHI) and calculated by summing across the squared market share of each firm in a market for all 50 states plus the District of Columbia. State-level HHI values were aggregated to the federal level by weighting according to state intercensal and total state population estimates for 2001 to 2016 from the U.S. Census Bureau [11,12]. Market concentration was then classified using HHI thresholds from the federal Horizontal Merger Guidelines: markets with HHI below 1,500, between 1,500 and 2,500, and above 2,500 were classified as unconcentrated, moderately concentrated, and highly concentrated, respectively [13].

## Results

Increased growth in direct written premiums from 2001 to 2016

**Figure 1:**
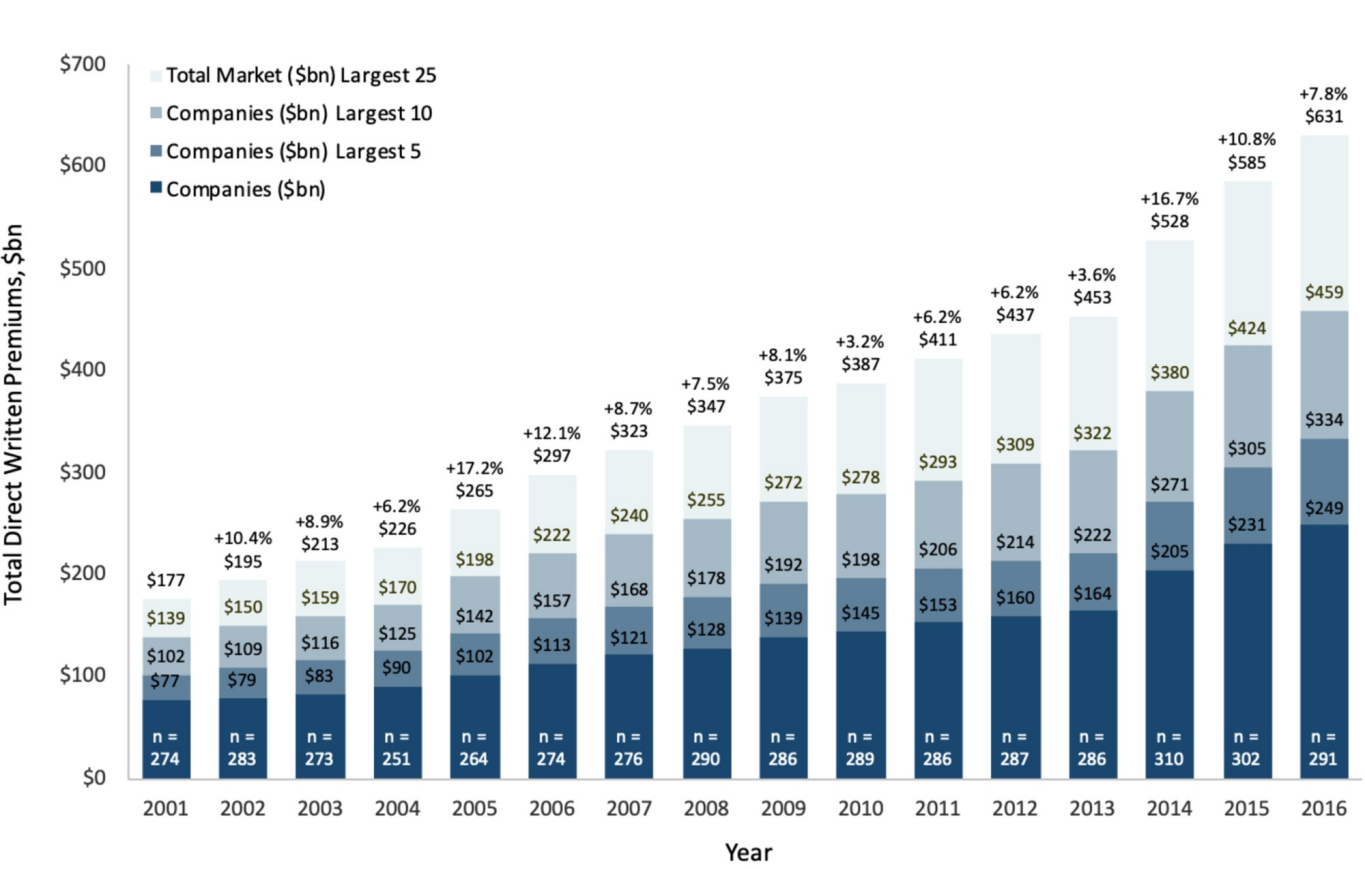
Direct written premiums and total market and largest companies by revenue (billion dollars), 2001-2016

Total DWPs for all health insurance companies increased rapidly between 2001 and 2016, growing nearly four-fold from $177 billion to $631 billion or at a compounded annual rate of 8.8% (Figure 1). Year-on-year growth in total DWPs was slowest in 2010 (3.2%) and 2013 (3.6%), and fastest in 2005 (17.2%) and 2014 (16.7%).

Total DWPs increased in all states between 2001 and 2016, as well as most states during the latest five-year period, 2011 to 2016, with the exception of New York. Total DWPs rose at the fastest compounded annual rate during the latest five-year period in Mississippi, Kentucky, Kansas, Louisiana, and West Virginia. Over the same five-year period, they rose at the slowest compounded annual rate in New York, California, Vermont, Pennsylvania, and Massachusetts. The total number of health insurance firms operating in the U.S. and reporting positive income grew from 274 in 2001 to 291 in 2016 (Figure 1). This value grew at the fastest rate over one year between 2013 and 2014 - at which point 310 firms were operational - before decreasing through 2016.

Overall, market share corresponding to the largest U.S. health insurance companies by total DWPs decreased from 2001 to 2016. This trend has more recently stabilized, with market share for the highest-grossing companies rising slightly between 2011 and 2016 (Figure 1), potentially owed to the resolution of the 2008 Great Recession financial crisis.

Decreased concentration of the U.S. health insurance market from 2001 to 2016

**Figure 2:**
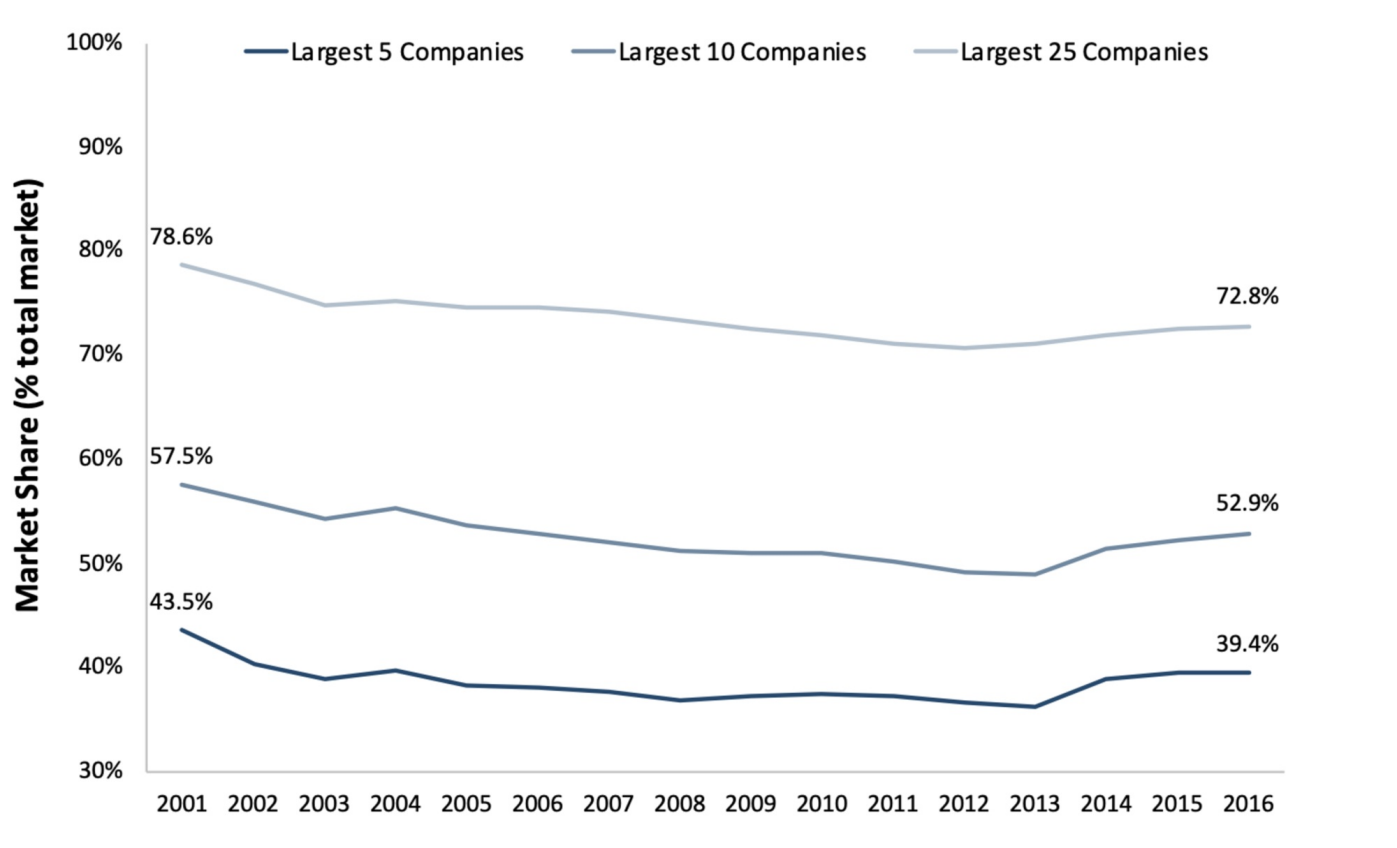
Market shares for the largest U.S. health insurance companies by revenue (% of the total market share), 2001-2016

From 2001 to 2016, the health insurance market did not undergo significant consolidation. The market share of the five largest healthcare insurance companies as measured by total DWPs, for instance, grew from 12% in 2001 to almost 40% in 2016. Appreciable jumps in market growth and consolidation were observed in 2004 and 2013. Nevertheless, the top 5, 10, and 25 publicly traded health insurance companies by market capitalization were 43.5%, 57.5%, and 78.6% of the total market share in 2001 and 39.4%, 52.9%, and 72.8% in 2016, respectively (Figure 2).

**Figure 3:**
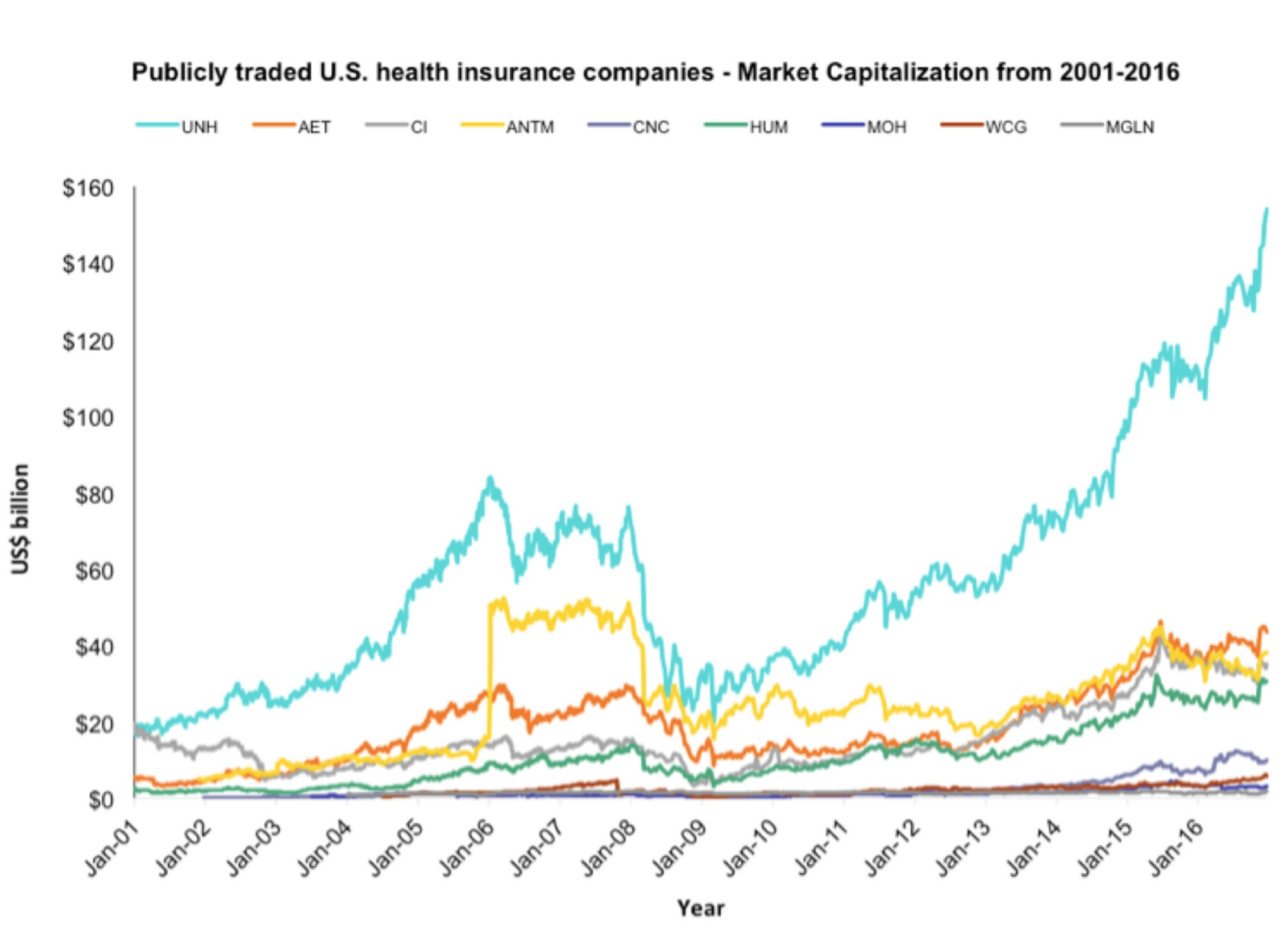
Market capitalizations (billion dollars) from 2001 to 2016 for the nine publicly traded U.S. health insurers UNH, UnitedHealth Group Incorporated; AET, Aetna Inc.; ANTM, Anthem Inc.; CI, Cigna Corporation; HUM, Humana Inc.; CNC, Centene Corporation; MOH, Molina Healthcare, Inc.; WCG, WellCare Health Plans, Inc.; MGLN, Magellan Health, Inc.

Of note, despite slowdowns in the immediate aftermath of the 2008 financial crisis, total market capitalizations have increased for the nine publicly traded U.S. health insurers since 2001 (Figure 3).

**Figure 4:**
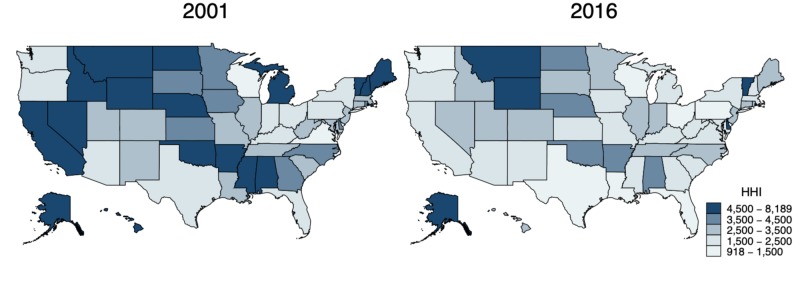
HHI of health insurance market concentration, state level, 2001 and 2016 HHI, Herfindahl-Hirschman index

Overall market concentration, as measured by HHI, has decreased since 2001. Population-weighted HHI values corresponding to the entire U.S. market fell from 3,817 to 2,174 between 2001 and 2016, with declines observed across most states (Figure 4). Long-term health insurance market concentration trends varied widely across states. Over the last five-year period of 2011-2016, HHI measures of market concentration decreased in 42 states, falling at the fastest rate in California (7,133 to 2,289), Iowa (6,104 to 2,434), Louisiana (3,364 to 1,494), Maine (5,768 to 2,609), and New Hampshire (5,372 to 2,442). HHI values increased in nine other states over the same period, rising at the highest rate in New Mexico (1,946 to 2,341), Delaware (4,480 to 5,303), Nevada (3,148 to 3,458), Vermont (6,036 to 6,570), and Connecticut (2,728 to 2,913). HHI declined more rapidly during 2011 to 2016 than during 2001 to 2010 in 35 of 51 states. HHI declines decelerated in the remaining 16 states: Arkansas, Colorado, Connecticut, District of Columbia, Delaware, Hawaii, Maryland, Michigan, Minnesota, Montana, New Mexico, Nevada, Oklahoma, Utah, Vermont, and Wisconsin (Figure 4).

Market concentration also remained high in many states. Seven state markets were unconcentrated in 2016 based on HHI values. A further 19 were moderately concentrated, whereas the remaining 25 were highly concentrated [13].

**Figure 5:**
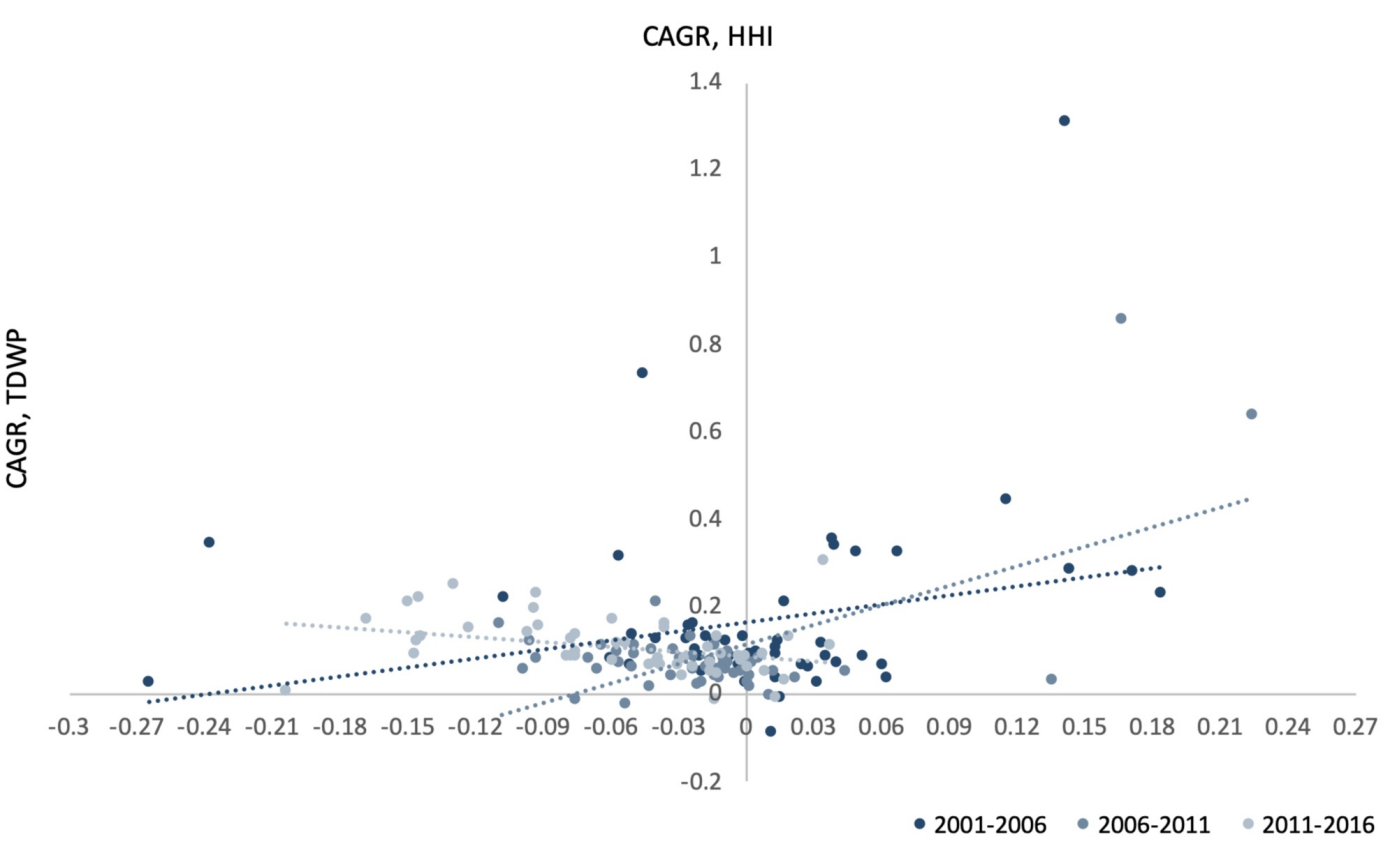
Correlation between compound annual change in HHI and TWDP, over the five-year periods (2001-2006, 2006-2011, and 2011-2016) CAGR, compound annual growth rate; HHI, Herfindahl-Hirschman index; TDWP, total direct written premiums

Compounded annual changes in state-level total DWPs over the five-year periods of 2001 to 2006 and 2006 to 2011 were positively correlated with state-level compounded annual changes in HHI measures (Figure 5). In other words, for the five-year periods of 2001 to 2006 and 2006 to 2011, the greater the state-level shift toward a more competitive health insurance market, the smaller the increase in total DWPs collected by insurers in those states. That trend, however, reversed over the latest five-year period, 2011 to 2016, as compounded annual changes in HHI measures also became less extreme (Figure 5).

## Discussion

This study used a unique financial dataset to analyze the U.S. healthcare insurance market and better understand how market concentration has changed with the emergence of state-level health insurance markets and affected total DWPs. Overall, total DWPs in the U.S. health insurance market grew at a compounded annual rate of 9% between 2001 and 2016, exceeding historical rates of healthcare inflation and population growth. They increased for each state in our analysis between 2001 and 2016 and for most states over the latest five-year period, 2011-2016, with the exception of New York and Connecticut.

However, growth in total DWPs has been uneven across U.S. states and over the time period considered. For instance, total DWPs grew at a compounded annual rate of 8% between 2001 and 2010 in New York before decreasing between 2011 and 2016 (compound annual growth rate: -1%). In contrast, growth in total DWPs has consistently been high in Mississippi, which witnessed the fourth highest compounded annual rate of growth in total DWPs among all states between 2001 and 2010 (39%) and the second highest rate of annual growth between 2011 and 2016 (25%). Growth in total DWPs nevertheless decelerated in a majority (27) of U.S. states during the latest five-year period. Additional research is needed to investigate the causes of variable growth across U.S. states in total DWPs collected by U.S. health insurers.

Market share corresponding to the largest publicly traded U.S. health insurance companies decreased between 2001 and 2016 (Figure 2), as did mean HHI measures of market concentration for the entire U.S. market. These trends were observed alongside a decrease in the concentration of most (42) state-level health insurance markets between 2001 and 2016. HHI measures of market concentration also decreased at a faster annual pace in a majority (35) of U.S. states between 2011 and 2016 than between 2001 to 2010. This coincided with the largest increase in the number of operational U.S. health insurance firms reporting any positive income over the entire 2001 to 2016 period, as well as with the implementation of the ACA.

There were several limitations to our study. While S&P Financial covers 99.9% of the world’s market capital and more than one million transactions annually, it is ultimately restricted to information on publicly traded companies and information voluntarily disclosed by privately-held companies. It fails to capture non-profit and mutual insurance companies, which can play important roles in individual healthcare markets depending on the state [14]. Second, under Medicare Advantage and Managed Medicaid, the scope of premiums has been greatly expanded; however, insurers do not necessarily offer coverage in the traditional group market [15,16]. Nevertheless, our paper is the first to use this novel Wall Street database to provide a different perspective from that of publicly traded healthcare insurance companies to reach similar conclusions to current studies, namely that at an aggregate level, the U.S. health insurance market has become less concentrated over the past decade [8].

Long-term trends in health insurance premiums and market concentration nevertheless vary across U.S. states. Despite an increase in the number of operational insurers and greater market competition, U.S. health insurance premiums have nevertheless continued to grow rapidly. As was especially evident for the latest five-year period, 2011-2016, increased competition in state-level health insurance markets also does not necessarily correlate with mitigated rises in total DWPs. Additional research is needed to examine how the creation of HIMs has affected competition between health insurers, how this has impacted costs for coverage, and the factors contributing to state-level differences in market concentration and premium collection trends.

As health insurance costs continue to place a growing burden on American families, our findings point to the need for greater efforts to study the impact on choice, quality, access, cost, and value to patients and providers from evolving health insurance markets.

## Conclusions

As measured by the market share of the top 5, 10, and 25 healthcare insurance companies, the market concentration of the publicly healthcare insurance market has remained relatively stable over the past decade. This has been accompanied by a relative stable growth in DWPs. As health insurance costs continue to place a growing burden on American families, our findings highlight the need for greater efforts to study the impact on choice, quality, access, cost, and value to patients and providers from evolving health insurance markets.
